# Insight into model mechanisms through automatic parameter fitting: a new methodological framework for model development

**DOI:** 10.1186/1752-0509-8-59

**Published:** 2014-05-20

**Authors:** Kristin Tøndel, Steven A Niederer, Sander Land, Nicolas P Smith

**Affiliations:** 1 Department of Biomedical Engineering, King’s College London, St. Thomas’ Hospital, Westminster Bridge Road, London SE1 7EH, UK; 2 Simula Research Laboratory, Martin Linges v. 17/25, Rolfsbukta 4B, Fornebu 1364, Norway

**Keywords:** Parameter estimation, Multivariate metamodelling, Parameter space exploration, Zooming into feasible parameter space regions, Experimental design, Model reduction, Computational Biology, Cardiac contraction

## Abstract

**Background:**

Striking a balance between the degree of model complexity and parameter identifiability, while still producing biologically feasible simulations using modelling is a major challenge in computational biology. While these two elements of model development are closely coupled, parameter fitting from measured data and analysis of model mechanisms have traditionally been performed separately and sequentially. This process produces potential mismatches between model and data complexities that can compromise the ability of computational frameworks to reveal mechanistic insights or predict new behaviour. In this study we address this issue by presenting a generic framework for combined model parameterisation, comparison of model alternatives and analysis of model mechanisms.

**Results:**

The presented methodology is based on a combination of multivariate metamodelling (statistical approximation of the input–output relationships of deterministic models) and a systematic zooming into biologically feasible regions of the parameter space by iterative generation of new experimental designs and look-up of simulations in the proximity of the measured data. The parameter fitting pipeline includes an implicit sensitivity analysis and analysis of parameter identifiability, making it suitable for testing hypotheses for model reduction. Using this approach, under-constrained model parameters, as well as the coupling between parameters within the model are identified. The methodology is demonstrated by refitting the parameters of a published model of cardiac cellular mechanics using a combination of measured data and synthetic data from an alternative model of the same system. Using this approach, reduced models with simplified expressions for the tropomyosin/crossbridge kinetics were found by identification of model components that can be omitted without affecting the fit to the parameterising data. Our analysis revealed that model parameters could be constrained to a standard deviation of on average 15% of the mean values over the succeeding parameter sets.

**Conclusions:**

Our results indicate that the presented approach is effective for comparing model alternatives and reducing models to the minimum complexity replicating measured data. We therefore believe that this approach has significant potential for reparameterising existing frameworks, for identification of redundant model components of large biophysical models and to increase their predictive capacity.

## Background

Models in computational biology are becoming increasingly complex, as in-silico frameworks are expanded to account for our rapidly increasing knowledge of physiological mechanisms [1]. This poses considerable challenges for uniquely linking model parameters to experimental data. The desire to capture this complexity to simulate physiological function increasingly results in models where the identifiability of parameters from available experimental data is relatively low. This situation is exacerbated by the lack of consensus on the optimal method for fitting model parameters to data, taking into account the, often, poor signal to noise ratio in these measurements. Furthermore, in many cases the model structure is such that the inverse problem of parameter fitting is ill-posed due to multiple parameter values producing the same model output. Finally, measured data in the literature is often incomplete, and state-of-the-art models are consequently based on a synthesis of data measured at different temperatures, from different laboratories and often from different species [2,3].

The reuse, combination and extension of existing models are necessary components of the Physiome approach [4]. In particular, as new datasets become available, and as models are applied to address new hypotheses and understand physiological situations, model developers are likely to need to augment or extend models or model components. This implies a requirement for a methodology for comparing model predictions with experimental data in a robust and automated fashion, efficiently incorporating new knowledge to better constrain the model parameters, systematically searching for the perturbation of the system that highlights parameter sensitivities and constrains the system, as well as reducing models to the minimal applicable version (as few parameters and equations as possible).

We believe that reduction in model complexity is important in that it typically increases the sensitivity of model outputs to the various parameters and hence the consequences of introducing changes to the model become more transparent. It also improves the likelihood that the models will be predictive outside the regime of the parameterising data. Specifically, if the identifiability of model parameters can be increased, this will enhance the ability to find the most relevant experimental measurements to use in order to constrain parameters within a given model framework, decreasing the uncertainty in parameter estimates.

In this study we address the issue of ill-posed inverse problems through the development of a generic framework for combined model parameterisation, comparison of model alternatives and analysis of model mechanisms. The fitting of model parameters from measured data is based on a combination of inverse metamodelling [5-9] (prediction of the input parameters as functions of the model outputs using regression) and iterative cost-function-based identification (look-up) of the simulations resulting in values of the output metrics in close proximity of the measured values, and subsequent zooming into relevant regions of the parameter space. In contrast to conventional nonlinear fitting or minimisation algorithms that only estimate parameter values, this method provides an overview of the parameter space and identifies regions in the parameter space where model outputs match measured data. The variation in possible solutions thereby provides an estimate of the uncertainty in the parameter values. Moreover, the inverse metamodelling component of the fitting pipeline provides an implicit sensitivity analysis and a quantification of the identifiability of model parameters from measured data.

In the look-up component of our proposed pipeline, the output spaces of model alternatives are analysed using Principal Component Analysis (PCA) [10,11], providing effective visualisation of the consequences of introducing changes to models and allowing identification of redundant model components. Hence, this modelling framework represents a combined parameter fitting and systematic analysis of model behaviour and model mechanisms for possible model reduction. This has the clear advantage that it provides a transparent link between parameter values and experimental data in comparison to alternative methods such as simplex optimisation [12], simulated annealing [13] and Levenberg-Marquart optimisation [14], which only provide parameter value estimates without increasing the understanding of the underpinning model mechanisms.

We demonstrate our proposed approach by applying our parameter fitting pipeline to re-parameterise the cardiac cell contraction model developed by Niederer *et al.*[15], originally fitted to rat experimental data at room temperature, to represent mouse functionality at 37°C by iteratively matching the output from the Niederer-model to a combination of measured data and the outputs of the Land-model [16] (which was parameterised for mouse at 37°C). The lack of a complete and self consistent data set of all output metrics of interest from a single species, temperature and laboratory motivated the use of simulated outputs from one model as a substitute for measured data in the parameter fitting. Using *in silico* data also provides the opportunity to analyse how the parameter identifiability can be increased by introducing additional output metrics for which measured values are not available in the literature, guiding future measurements.

Following re-parameterisation of the Niederer-model, we apply the same methodology for finding reduced model versions through the identification of redundant model components. Specifically, we demonstrate how our methodology can be used for systematically comparing model versions, analysing the sensitivity of the model outputs to the input parameters, and choosing the most reduced version giving outputs matching measured data.

## Methods

### Application system

As outlined above we demonstrate our methodology by applying it to two models of cardiac cell contraction, consisting of differential equations describing contractile force, including length-dependence and velocity-dependence. The choice of application system was motivated by the high degree of maturity of cardiac models; the heart is arguably the most advanced example of a multi-scale framework for biology. Both these models represent cardiac muscle cells which consist of many contractile sub-units, sarcomeres, each again organised into thin and thick filaments [17,18]. The thick filaments contain myosin crossbridges that bind to the thin actin filament to generate force. Electrical activation results in an increase in cytosolic calcium (Ca), and binding of calcium to the regulatory calcium binding site on troponin C (TnC) within the sarcomeres. This causes a conformational change in the associated tropomyosin complex that unblocks the thin filament actin sites for binding to the thick filament myosin crossbridges. In a crossbridge cycle, a myosin crossbridge on the thick filament attaches to the actin thin filament, performs a power stroke to generate force, and then detaches using Adenosine Triphosphate (ATP). Both models applied in this study consist of equations representing the influence of the muscle’s length on the tension it generates (length-dependence; more force is generated as a muscle is stretched), and the sensitivity of the generated force to the rate at which the muscle is stretched (velocity-dependence). The velocity-dependence parts of the two models are based on the same mathematical formulation, which is therefore not considered in this study (the velocity was set to zero for all simulations). Both models, parameterised from a range of data, are biophysically based, and represent two different frameworks for simulating the generation of contractile force in cardiac cells as a consequence of calcium binding (a central component of heart physiology). A description of the two contraction models including the differential equations is given in Additional file 1.

Both the Land-model and the Niederer-model were developed specifically for use with organ-scale simulations, and therefore have a relatively low level of detail compared to many other contraction models. Specifically, they do not include many sub-states for the attachment of ATP and the position of crossbridges. However, both of these models do include enough detail to enable the direct linking of parameters to biological data and exploration of different mechanistically based hypotheses.

The Niederer-model was originally parameterised using data for rats at 25°C, the calcium/TnC dynamics are modelled by a simple molecular binding model, and tropomyosin/crossbridge dynamics are represented by the transient changes in the proportion of available actin sites, while the binding sensitivity is length-dependent. With the default parameter values, the Niederer-model is unable to capture the fast relaxation kinetics of mouse cardiac muscle at higher pacing frequencies.

The Land-model uses a standard cooperative binding equation which has a Hill curve as its steady state solution to represent troponin binding, where the calcium half activation of maximal steady state tension generation is length-dependent, combined with a modified version of the crossbridge dynamics component from the model developed by Rice *et al.*[19], which uses a 4-state Markov model. The Land-model uses only 2 of these states, the so-called non-permissive and permissive (crossbridge cycling) states.

Evidence of the velocity-dependence of tension generation and the dynamic response to step changes remains controversial in the experimental literature. The fading memory model (FMM) [20] provides a succinct representation of these dynamics without being tied to a specific underlying mechanism, and is exploited by both models. The FMM represents the velocity response as several strain-rate dependent variables which all decay with time. An advantage of this model is that it is independent of the contraction model, and can be added after modelling isometric tension and length-dependence.

Our analysis of the two contraction models consists of the following steps:1) Sensitivity analysis and parameter identifiability analysis based on statistically designed model simulations and metamodelling. This was carried out to test whether the Niederer-model parameters could be predicted directly from the Land-model outputs using regression, and to identify redundant model components for both models. This is illustrated in Figure 1.2) Due to the relatively low prediction accuracy of the resulting inverse metamodel for several of the Niederer-model parameters, the inverse metamodelling approach was combined with a cost-function based look-up of simulations resulting in model outputs in close proximity to the target values. This was carried out iteratively as shown in Figure 2, resulting in a zooming into the region of the parameter space where the target outputs were replicated by the simulations.

**Figure 1:**
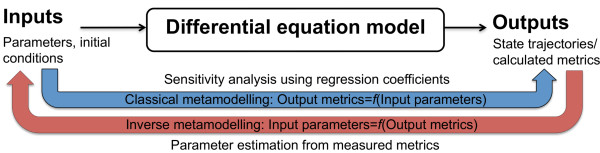
Illustration of classical and inverse metamodelling for sensitivity analysis and parameter estimation.

**Figure 2:**
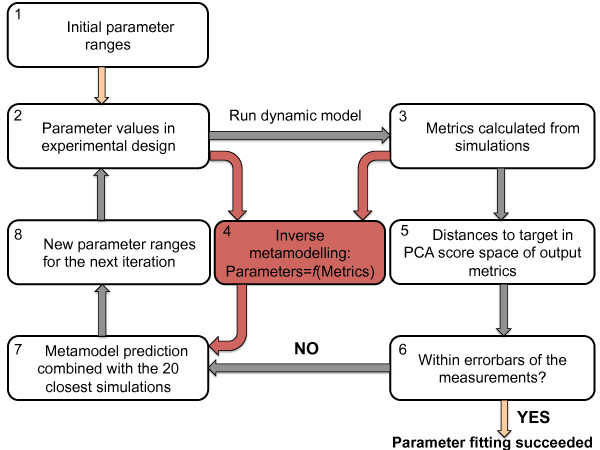
**Schematic representation of the parameter fitting pipeline.** Steps 2–8 were repeated in each iteration.

3) Model reduction by repetition of step 2 using reduced model versions. The reduced model versions were made based on the results from the parameter identifiability analysis, which was done for both models.

### Sensitivity analysis and parameter identifiability analysis of the Niederer-model

**Figure 3:**
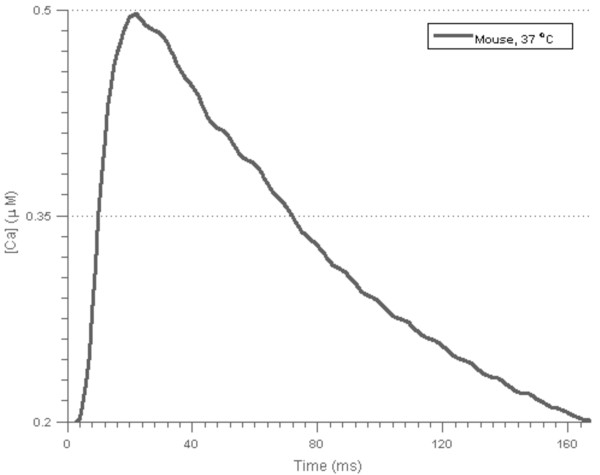
**The measured Ca-transient used in all simulations with the two contraction models.** The transient was measured for mouse at 37°C.

In order to obtain an overview of the relationships between input parameters and dynamic outputs of the model, an experimental design of the Niederer-model parameters using relatively wide parameter ranges was made using a Latin Hypercube design (LHD) [21]. LHD is a semi-random sampling procedure that is especially suitable for use on high-dimensional data, since it separates the data into several hypercubes, and samples randomly within each hypercube. This ensures that all regions of the parameter space are sampled. Within our implementation, the parameter ranges in Table 1 were used to generate a LHD of 500 parameter value combinations, and simulations where run with the Niederer-model using cell lengths of 90, 100 and 110% of resting sarcomere length. An input Ca-transient measured for mouse at 37°C (Figure 3) [22] was used in all simulations. All simulations and subsequent analyses were done in MATLAB^®^ version R2012b [23].

**Table 1:** Description and initial ranges for the varied Niederer-model parameters

**Parameter**	**Description**	**Minimum value**	**Maximum value**	**Default value**
*Ca*_*50ref*_	Calcium sensitivity at resting sarcomere length (mM)	0.3e-3	0.8e-3	0.3e-3
*k*_*refoff*_	Unbinding rate of Ca from TnC in the absence of tension (ms^-1^)	0	0.80	0.2
*k*_*on*_	Binding rate of Ca to TnC (μM^-1^ s^-1^)	0	400	100
*n*_*r*_	Relaxation parameter	2	4	3
*β*_*0*_	Magnitude of length-dependent activation effects	1	5	4.9
*β*_*1*_	Magnitude of filament overlap effects	-8	0	-4
*γ*	Effect of tension on the unbinding rate of Ca from TnC	2	100	2
*nH*	Hill coefficient in the steady-state force-pCa curve	4	9	5
*T*_*ref*_	Reference tension (kPa)	100	140	100
*α*_*0*_	Monoexponential activation rate seen in caged Ca experiments (ms^-1^)	0	0.048	0.008
*α*_*r1*_	Slow relaxation rate (ms^-1^)	0	0.012	0.002
*α*_*r2*_	Fast relaxation rate (ms^-1^)	0	0.0105	0.00175
*K*_*z*_	Relaxation parameter	0.1	0.2	0.15

#### ***Output metrics used to represent the model behaviour***

Tension transients were simulated using both the Land and Niederer contraction models, and described by routinely experimentally measured descriptors of the transient morphology. A list of the descriptors and their recorded experimental values for mouse at 37°C is shown in Table 2. Tension transients were simulated at three cell lengths (90, 100 and 110% of resting sarcomere length) activated by the experimentally measured Ca-transient in Figure 3.

**Table 2:** **Metrics used to describe the tension transients and measured data for mouse at 37**°C

**Metric**	**Description**	Land-model default output^*^	**Experimental values**
*RT50*	Time to 50% relaxation (ms)	24	16-30
*RT90*	Time to 90% relaxation (ms)	53	41-59
*TTP*	Time to peak tension (ms)	33	26-41
*Peak*	Peak tension (kPa)	41.1	32-52
*Min*	Minimum tension (kPa)	0.073	

These metrics were calculated from simulations using a Ca-transient.

^*^From a simulation at resting sarcomere length.

Preliminary analyses of the results achieved by fitting the model parameters to the metrics in Table 2, using data obtained by simulations using the experimentally measured Ca-transient scaled by 90, 100 and 110%, showed that the model outputs were highly sensitive to the calcium concentration. In order to take this into account we also matched the force-pCa (*F*-*pCa*) relationships of the two models, using metrics from simulations run with fixed Ca-concentrations as additional model characteristics to constrain parameters. The Ca-concentrations used were a logarithmically spaced series of 82 different concentrations from 0.15 to 1 μM together with the concentration 10 μM. The resulting steady state tensions were normalised by the maximal simulated tension value.

Model and experimental steady state force-calcium curves are routinely approximated by a Hill-curve that can be logarithmically transformed to be linear. The relationship between *pCa* and log(*F*/(1-*F*)) was therefore fitted to a straight line using Ordinary Least Squares (OLS) Regression [24] (values of (1-F) < 10^-3^ were removed in order to avoid numerical errors), and the metrics given in Table 3 were calculated to represent the properties of the force-pCa relationship. The *F*-*pCa* curves were simulated for 90, 100 and 110% of resting sarcomere length, and the resulting *F*-*pCa* metrics used as additional output constraints (together with the tension transient characteristics resulting from simulations with the experimental Ca-transient) to fit the parameters of the Niederer-model. Similarly, the final set of target output measures included both the metrics in Table 2 and those in Table 3, all calculated from simulations with 90, 100 and 110% of resting sarcomere length for the Land-model.

**Table 3:** Description of the output metrics used to describe the force-pCa relationship

**Metric**	**Description**	Land-model default output^*^
*Slope*	Slope of the fitted line	-7.33
*Intercept*	Intercept of the fitted line	45.9
*RMSEP*	Root Mean Square Error of prediction from fitting to a straight line (representing the deviation from a straight line)	0.18
*R*^*2*^*force*	Correlation coefficient between the fitted line and the simulated force-pCa data (representing the deviation from a straight line)	0.99
*Max*	Maximum tension	119.4
*RMSDforce*	RMSD between the simulated force for the Niederer-model and the target Land-model force (in standardised variables)	0

These metrics were calculated from simulations using fixed Ca-concentrations.

^*^From a simulation at resting sarcomere length.

#### ***Sensitivity analysis by classical metamodelling***

Partial Least Squares Regression (PLSR) [25-28] was then used for regression-based sensitivity analysis. PLSR is a subspace-based regression method based on decomposing the data into a subspace representing the main features of covariance between the regressors (here input parameters) and the response variables (here model output metrics). This subspace is represented by latent variables called score- and loading vectors. PLSR can be seen as a regression analogue to PCA, and can handle numerous input and output variables simultaneously. Unlike for example OLS Regression [24], PLSR does not require the regressor variables to be linearly independent. Coupling between parameters can be revealed using PLSR-based classical metamodelling through analysis of the regression coefficients for cross-terms between the parameters. In addition, the correlations (Pearson’s R) between all input parameters and output metrics included in the analysis were evaluated to obtain overview of the model system.

Based on the parameter-simulated output data for the Niederer-model, a classical metamodel was first constructed to predict the output metrics as functions of the parameters using PLSR. This classical metamodel was used for sensitivity analysis, using the regression coefficients as sensitivity measures (measures of the impact of variations in each of the parameters on the output metrics), as described in [29,30]. The metamodelling procedure is illustrated schematically in Figure 1. Here, both parameters and output metrics were centred and standardised by subtraction of the mean value and dividing by the standard deviation of each variable prior to the regression, making the regression coefficients independent of the scales of the variables and thereby easier to compare in the sensitivity analysis. Cross-terms and second order terms of the input parameters (i.e. products between combinations of variables in the regressor matrix) were included in the metamodelling to take nonlinearities into account when predicting the output metrics.

#### ***Parameter identifiability analysis by inverse metamodelling***

To evaluate whether it would be possible to get a reasonable estimate for the Niederer-model parameters by direct prediction using regression, an inverse metamodel, predicting the input parameter values from the simulated output metrics in Table 2 and Table 3, was generated using Hierarchical Cluster-based Partial Least Squares Regression (HC-PLSR) [5,6]. HC-PLSR is a nonlinear extension of PLSR. As described above, PLSR can handle correlated regressor variables, which makes it especially useful for inverse metamodelling of large, complex dynamic models, which contain large sets of highly coupled differential equations producing correlated model outputs. HC-PLSR is a locally linear regression method based on separating the observations into groups using fuzzy *C*-means (FCM) clustering [31-34] on the latent variables from a global PLSR model including all observations, and making local PLSR models within each cluster. This allows prediction of highly nonlinear input–output relationships. The inverse metamodelling procedure is also schematically illustrated in Figure 1, while the HC-PLSR method used for the metamodelling is outlined in Additional file 2.

Both parameters and output metrics were centred and standardised by subtraction of the mean value and dividing by the standard deviation prior to the regression, and 8 clusters where used in the HC-PLSR. The number of clusters was chosen based on a comparison of predictive ability between different HC-PLSR metamodel complexities ranging from models using 1–20 clusters. This comparison showed that 8 clusters was the minimum number of clusters providing maximal predictive ability, and 8 clusters were therefore used to limit the metamodel complexity. Cross-terms and second order terms of the output metrics were included in the inverse metamodelling to predict the input parameters, in order to better handle nonlinearities in the input–output relationships of the model.

Due to the relatively large differences between the default outputs from the Land-model and the Niederer-model, it was necessary to obtain a robust estimate of the predictive ability of the metamodel to evaluate whether it could be used to directly predict new parameter values for the Niederer-model. The inverse metamodel was therefore validated using 33% of the simulations from the experimental design of 500 simulations as a separate test set. Hence, the metamodel was calibrated using only 2/3 of the simulations, while the rest of the simulation results were kept aside for the purpose of prediction and thus not included in the parameterisation of the metamodel. This process produces a valid estimate of the ability of the metamodel to predict the parameter values from a new set of measured data.

### Fitting of the Niederer-model parameters

The results from the sensitivity analysis and the parameter identifiability analysis above showed that the identifiability was relatively low for several of the Niederer-model parameters (see the Results section). We therefore combined the inverse metamodelling with an iterative generation of new experimental designs in the parameters, and identification of the simulations resulting in output metrics in close proximity to the target values. The target output metrics were found from simulations run with the Land-model using the default parameter set and otherwise the same settings as for the Niederer-model simulations. These were used as substitutes for measured data in the parameter fitting pipeline. A schematic representation of the parameter fitting pipeline is shown in Figure 2. The initial Niederer-model parameter ranges are given in Table 1, and were used to generate the initial experimental design (step 2 in Figure 2). Following simulations with the contraction model, the output metrics described above were calculated from the model outputs generated using the parameter values from the experimental design (step 3).

The next step of the fitting pipeline is to generate an inverse HC-PLSR metamodel, predicting the Niederer model parameters as functions of the output metrics in Table 2 and Table 3, based on the simulation results. This metamodel is then applied to the target outputs (from the Land-model simulations, see Table 2 and Table 3) to generate an initial estimate of the model parameters (step 4 in Figure 2). The inverse metamodelling is performed in the same way as described above under “Parameter identifiability analysis by inverse metamodelling”.

For each set of output metrics corresponding to one of the parameter sets in the experimental design, the proximity to the target is found (step 5), and the predicted parameter set from the inverse metamodelling is then combined with the 20 simulations that generated observations in the closest proximity to the experimental measurements (step 7). The predictions from the metamodelling were only included for those parameters for which the inverse metamodel could provide a prediction accuracy of >70% in a test set validation. Together, these 21 parameter sets (in the following referred to as the “guideline sets”) are used to identify the direction or localised region of the parameter space that allows the model to best match the target observations. Using the 20 simulations having the lowest distances to the measured metrics in the guideline set was considered sufficient in order to balance between zooming into the relevant parameter space region and keeping the possibility of identifying alternative regions giving feasible output metrics. This increases the likelihood that all possible regions in the parameter space that can produce physiologically feasible simulations are found during the parameter fitting. This, preferably together with constraints on the parameter values according to *a priori* knowledge about possible ranges, can generate robust/unique parameter estimates. The size and number of identified regions of the parameter space producing model outputs that replicate measured data give an indication of the uniqueness of the parameter estimates.

The achieved distances to the target outputs are found by PCA of the output metrics resulting from the simulations together with the target output (using centred and standardised variables), and calculation of the Root Mean Square Distances (RMSDs) of each simulation to the target in the PCA scores. The PCA scores are used to evaluate the distance to the target both in order to decrease the dimensionality of the data and to weight the metrics according to their contribution to the variation in the data. The PCA approach decomposes the data into latent variables describing the main variance directions in the data, and each score vector is a weighted sum of the original variables. Hence, the metrics having the largest contributions to the variation in the data have the highest weights for the first principal components (PCs) resulting from the PCA. The minimal number of PCs explaining 99% of the variance are included in the distance calculations in our fitting pipeline.

For each parameter, the new parameter range for the next iteration is set to the value span over the guideline sets (X1) ± an additional span defined by a variable called *stepsize*_*new*_ (in order to extend the design beyond the values for the guideline sets and thereby further approach the target output values (step 8 in Figure 2)). The ranges for the new experimental design are calculated using Equations (1) and (2).

(1) $$\text{Maximum}\;\text{values}\;\left(i\right)=\;{\text{max}}_{i}X1+\left|\left(\frac{\overset{¯}{X1_{i}}}{\text{stepsiz}e_{\text{new}}}\right)\right|$$

(2) $$\text{Minimum}\;\text{values}\;\left(i\right)=\;{\text{min}}_{i}X1-\left|\left(\frac{\overset{¯}{X1_{i}}}{\text{stepsiz}e_{\text{new}}}\right)\right|$$

The variable *stepsize*_*new*_ was introduced to allow adjustment of the spread in parameter values according to the degree of proximity to the target outputs. Initially, the value of *stepsize*_*new*_ is 4 in order to analyse a large part of the parameter space. In each following iteration, the minimum achieved RMSD in the PCA score space is compared to that for the previous iteration, and *stepsize*_*new*_ is increased by 2 if the value has decreased, until it reaches a maximum value of 20. Hence, the value of *stepsize*_*new*_ is increased as the distance from the target decreases, strengthening the zooming effect. If *stepsize*_*new*_ reaches the value 20 before the results are sufficiently close to the target metrics values, *stepsize*_*new*_ is decreased by 2 for the next iteration design.In each iteration, a new experimental design of parameter value combinations is generated using LHD in the region of the parameter space defined by the new parameter ranges. The number of simulations for each iteration is given as input to the fitting pipeline. Here, using a LHD size of 500 simulations was regarded sufficient in order to sample the parameter space relatively densely, while limiting the computational time used in each iteration. This procedure (step 2–8 in Figure 2) is repeated iteratively until the success criterion is met (evaluated in step 6 in Figure 2).

For our specific application, the criterion for success of the parameter fitting was defined as follows:

1) For resting sarcomere length simulations: The tension transient metrics should be within the error bars for the measurements in Table 2.

2) For 110% of resting sarcomere length simulations: The peak tension should be between 73 and 90 kPa (based on experimental measurements of relative changes in maximum twitch force generation [16]) and the minimum tension should be less than 1 kPa.

3) For 90% of resting sarcomere length simulations: The peak tension should be between 12 and 20 kPa (±20% of the baseline value from the Land-model).

4) For the force-pCa curve simulations: The RMSD between the simulated force for the Niederer-model and the target Land-model force (in standardised variables) should be less than 15%.

The test set prediction accuracy of the inverse metamodel was relatively low for several of the parameters (see the Results section), so the metamodel was used only in the first iteration to provide an initial indicator of the direction in the parameter space to move (by adding extra extensions to the ranges of some of the parameters based on the prediction). The constraints given in Table 4 were set on the parameters based on the variation in measured values in the literature.

**Table 4:** Constraints used on some of the Niederer-model parameters used in the parameter fitting

**Parameter**	**Minimum value**	**Maximum value**
*k*_*refoff*_	0.05	0.4
*k*_*on*_	50	300
*n*_*r*_	1	-
*β*_*0*_	-	6
*γ*	1	5
*nH*	1	15
*T*_*ref*_	90	140

The fitting pipeline was written in MATLAB^®^ version R2012b [23] as both a parallelised and a non-parallelised version, and both can be obtained from the authors upon request.

### Reduction of model complexity for the Niederer-model and the Land-model

#### ***Parameter identifiability analysis for the Land-model***

In the same way as for the Niederer-model, the possibility for reducing the Land-model was tested based on a similar test set validated inverse HC-PLSR metamodelling. The metamodel was made using data from simulations in a LHD of 500 observations within the ranges given in Table 5, using the output metrics in Tables 2 and 3 to predict the Land-model parameters. All variables were centred and standardised prior to the regression, and cross-terms and second order terms of the output metrics were included.

**Table 5:** Description and ranges for the Land-model parameters used for parameter identifiability analysis

**Parameter**	**Description**	**Minimum value**	**Maximum value**	**Default value**
*T*_*ref*_	Reference tension (kPa)	100	140	120
*Ca*_*50ref*_	Calcium sensitivity at resting sarcomere length (μM)	0.5	0.8	0.7
*TRPN*_*50*_	Troponin C sensitivity	0.25	0.5	0.35
*n*_*TRPN*_	Hill coefficient for cooperative binding of Ca to TnC	1	2.5	2
*k*_*TRPN*_	Unbinding rate of Ca from TnC (ms^-1^)	0	0.5	0.1
*n*_*xb*_	Hill coefficient for cooperative crossbridge action	3	7	5
*k*_*xb*_	Scaling factor for the rate of crossbridge binding (ms^-1^)	0	0.6	0.1
*β*_*1*_	Magnitude of length-dependent activation effects	-2	-1	-1.5
*β*_*0*_	Magnitude of filament overlap effects	1	5	1.65

#### ***Model reduction***

##### 

**Reduction of the Niederer-model** The parameter fitting procedure described above was repeated with parts of the Niederer-model omitted in order to see whether the model could be reduced while keeping the replication of the simulated output data from the Land-model. The choice of model parts to omit was based on the results from the sensitivity- and parameter identifiability analysis, indicating very low sensitivity to the parameters *n*_*r*_, *α*_*r2*_ and *K*_*z*_. These parameters were assumed to have minor effects on the model outputs, and were therefore omitted by making the model independent of these model parts. This omission was achieved by giving the parameter *α*_*r2*_ the value zero, making the model independent also of *n*_*r*_ and *K*_*z*_.

The initial parameter ranges in the fitting pipeline were the same as in the previous parameter fitting (given in Table 1), and all output metrics in Tables 2 and 3 were included to fit the model parameters.

##### 

**Reduction of the Land-model** Based on the parameter identifiability analysis of the Land-model, *k*_*TRPN*_, *n*_*xb*_ and *k*_*xb*_ were successively set equal to 1 (keeping the other parameters at the default values), in order to analyse the model output effects of variations in these parameters. The simulations were run as described above, and all output metrics in Tables 2 and 3 were included in the analysis.

## Results

As described in the Methods section, we have analysed the sensitivity of the model outputs to variations in the input parameters, verified parameter identifiability and compared and matched the model outputs for the two cardiac contraction models. The analyses were based on both simulations run using a measured Ca-transient for mouse at 37°C to generate dynamic tension transients, and fixed, individual Ca-concentrations to simulate the steady state *F*-*pCa* curve. The Niederer-model was re-parameterised using the presented parameter fitting pipeline in Figure 2 using a combination of measured data and synthetic data from Land-model simulations. Reduced versions of both models were identified based on the parameter identifiability analysis and comparison of the outputs from reduced model versions with the Land-model default outputs. The results are detailed below.

### Sensitivity analysis and parameter identifiability analysis of the Niederer-model

#### ***Sensitivity analysis by classical metamodelling***

**Figure 4:**
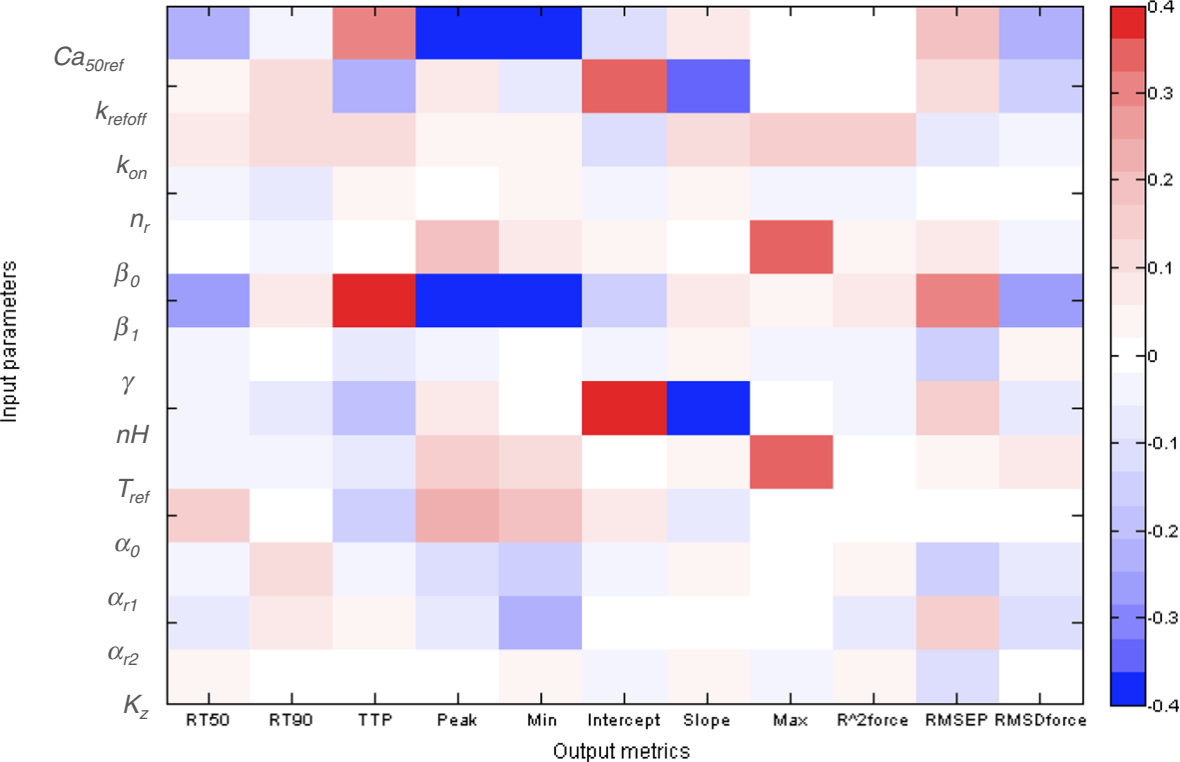
**Regression coefficients from the classical PLSR metamodel.** The regression coefficients were used to analyse the model sensitivities to the different input parameters. Results are shown for 110% of resting cell length.

**Figure 5:**
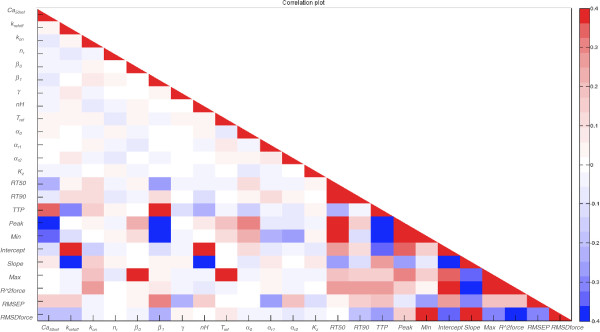
**Correlations (Pearson’s R) within and between input parameters and output metrics.** Results are shown for 110% of resting cell length.

Sensitivity analysis based on a classical PLSR metamodel indicated that physiological simulations using the Niederer-model had low sensitivity to the parameters *n*_*r*_, *γ*, *α*_*r2*_ and *K*_*z*_, while they were most sensitive to *Ca*_*50ref*_, *k*_*refoff*_, *β*_*0*_, *β*_*1*_, *nH* and *T*_*ref*_. The regression coefficients from the PLSR showing the sensitivity of the output metrics to the input parameters are shown in Figure 4. These results indicate that parts of the Niederer-model tropomyosin kinetics component can be simplified by omitting the low sensitivity parameters. The model equations in Additional file 1 show that giving *α*_*r2*_ the value zero would make also *n*_*r*_ and *K*_*z*_ redundant, significantly reducing the model complexity. This possibility was therefore analysed further below.The sensitivity patterns described above were confirmed by the plot of the correlations (Pearson’s R) between all input parameters and model output metrics included in this analysis, shown in Figure 5. As expected due to the sampling procedure used to generate the experimental design of parameter sets, Figure 5 shows no strong correlations between the input parameters in the model. However, there are several strong correlations between the output metrics. This was also expected, since they are metrics representing curve shapes. However, the results also show correlations between the metrics representing the tension transients and those representing the force-pCa-relationship.

#### ***Parameter identifiability analysis by inverse metamodelling***

**Figure 6:**
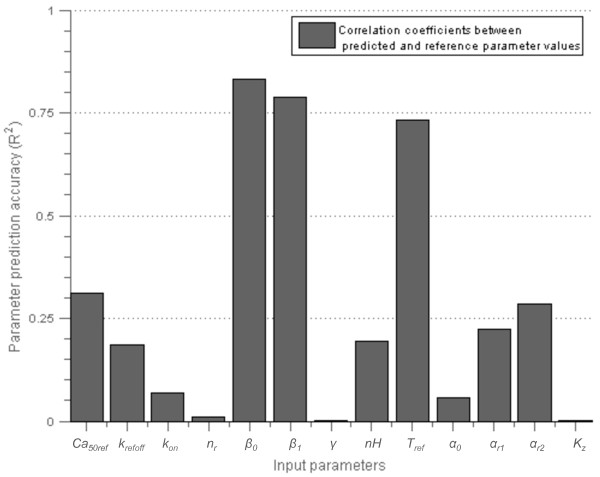
**Results from the parameter identifiability analysis of the Niederer-model.** Parameter prediction accuracies from the inverse HC-PLSR metamodel, which was test set validated using 33% of the simulations as an independent test set. 8 clusters were used in the HC-PLSR.

The parameter prediction accuracies from the inverse HC-PLSR metamodel are shown in Figure 6, represented by the correlation coefficients (*R*^*2*^-values) between the simulated and the predicted parameters from a test set prediction. The test set consisted of 33% of the simulations from the LHD of 500 simulations. These simulations were not included in the calibration of the metamodel, and therefore represent new data, so that the resulting predictive ability would be what we can expect from a prediction using new measured data (or the output from simulations with the Land-model). As Figure 6 shows, the inverse metamodel was not able to predict all parameters accurately, but could give useful information about the parameters *β*_*1*_, *β*_*0*_ and *T*_*ref*_. The reason why some of the model parameters that the sensitivity analysis indicated a model sensitivity to were predicted incorrectly by the inverse metamodel is probably that the model is sloppy, meaning that many parameter value combinations can generate the same output metrics values. This characterises most dynamic models [35]. The model can still be sensitive to variations in these parameters, but it is difficult to predict parameter values from the output metrics for sloppy models. However, our results demonstrate the value of using inverse metamodelling to give an indication of the best direction in the parameter space to move to approach reasonable estimates for the values of the three parameters for which the prediction accuracy was relatively high. For the other parameters the inverse metamodel alone will not provide an estimate that can be trusted. The fitting procedure therefore had to be extended by including the look-up approach to guide new simulations.

### Fitting of the Niederer-model parameters

**Figure 7:**
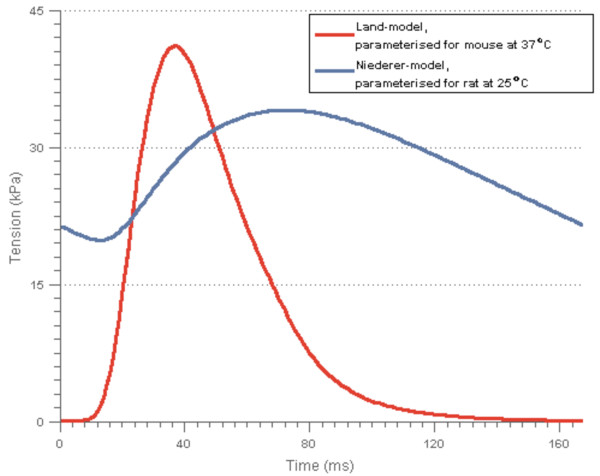
**Tension transients for the Land- and Niederer-models with default parameter values at resting cell length.** The transients were achieved through simulations using the Ca-transient shown in Figure 3. The Niederer-model was originally parameterised using a Ca-transient measured for rat at room temperature.

**Figure 8:**
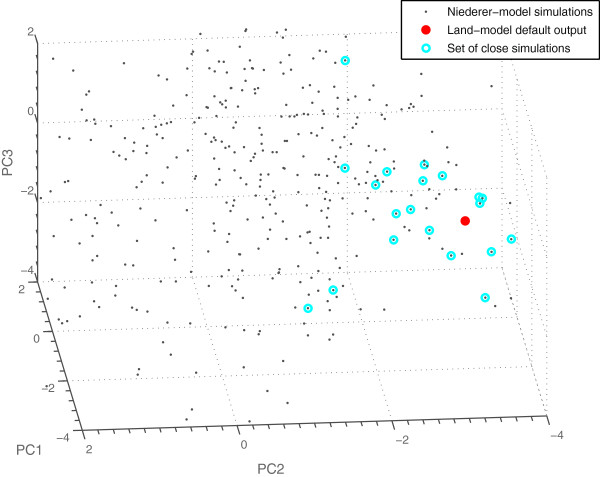
**Comparison of simulation results from the first iteration with the target output metrics.** Results from a PCA of the simulations based on Table 1 (grey), together with the results from the Land-model (red). The 20 simulations closest to the target are marked with circles in cyan. The corresponding 20 parameter sets were used together with the parameter prediction from the inverse metamodelling to find new parameter ranges and generate a new experimental design for simulations with the Niederer-model.

Figure 7 shows a comparison of the outputs from simulations with default parameter values and resting cell length for the two models. As the figure shows, the Niederer-model is not able to capture the faster relaxation kinetics of the mouse at higher pacing frequencies. This was expected, since the Niederer-model was originally parameterised using a different Ca-transient and fitted to experimental measurements from a different species. Figure 8 shows the results from a PCA of the simulation results based on the parameter value combinations generated by the initial experimental design using the parameter ranges in Table 1, together with the results from the Land-model. This was the PCA used in the first iteration of the fitting pipeline.

**Figure 9:**
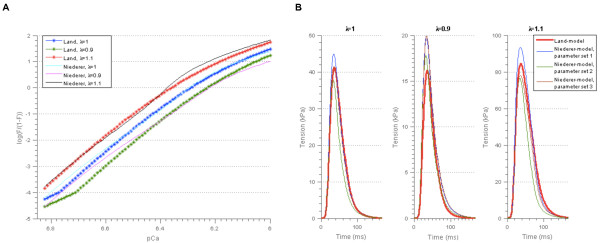
**Resulting model outputs after re-parameterisation of the Niederer-model. A)** Force-pCa relationship for parameter set 1 in Table 6. The force-pCa relationships for the remaining parameter sets in Table 6 are shown in Additional file 3: Figure A3.1. The parameter λ represents the cell length relative to the resting cell length. **B)** Tension transients for the three simulations for which the force-pCa relationships matched that of the Land-model.

**Figure 10:**
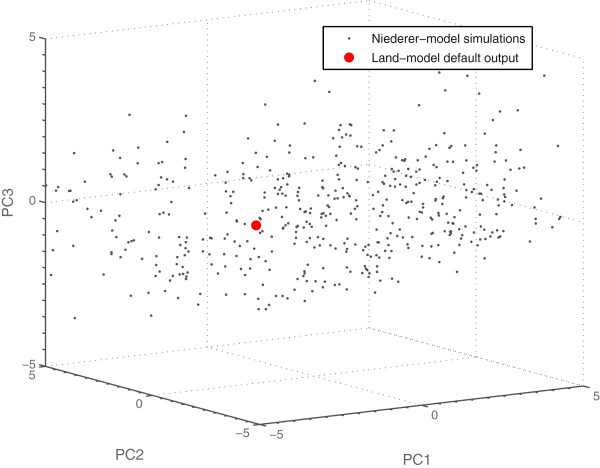
**Comparison of simulation results from the last iteration with the target output metrics.** PCA of the output metrics for the 500 simulations from the last iteration with the Niederer-model (grey) together with the Land-model default outputs (red).

Using our presented parameter fitting pipeline, three Niederer-model parameter sets were identified that fitted the Land-model data. The three successful parameter sets found (see Table 6) gave outputs from the Niederer-model matching the Land-model outputs for all three cell lengths used, including the force-pCa relationships. The force-pCa relationship for parameter set 1 in Table 6, which gave the best match to the Land-model outputs, and the tension transients for all parameter sets in Table 6 are shown in Figure 9. The force-pCa relationships for the remaining parameter sets in Table 6 are shown in Additional file 3: Figure A3.1. The spread in parameter values provides an indication of how constrained a parameter is for a given set of output metrics. In this specific case, the Niederer-model parameters could be constrained to a standard deviation of on average 17.4% of the mean values over the succeeding parameter sets. Figure 10 shows the 500 simulations from the LHD used in the last iteration together with the Land-model output in the score space from a PCA of all output metrics. As expected, the simulation results are significantly closer to the region of the Land-model outputs than in the first iteration (see Figure 8).

**Table 6:** Niederer-model parameter values corresponding to the model output values closest to the target

**Parameter**	**Parameter set 1**	**Parameter set 2**	**Parameter set 3**	**Mean value**	**Standard deviation**
*Ca*_*50ref*_	0.31e-3	0.33e-3	0.35e-3	0.33e-3	2.21e-5
*k*_*refoff*_	0.08	0.12	0.08	0.09	0.03
*k*_*on*_	227.2	163.1	186.7	192.3	32.4
*n*_*r*_	1.38	1.66	2.02	1.68	0.32
*β*_*0*_	0.10	0.06	0.07	0.08	0.02
*β*_*1*_	-1.35	-1.14	-1.29	-1.26	0.11
*γ*	3.99	4.75	4.79	4.51	0.45
*nH*	13.48	10.23	12.09	11.93	1.63
*T*_*ref*_	135.8	130.6	115.8	127.4	10.4
*α*_*0*_	0.03	0.03	0.06	0.04	0.02
*α*_*r1*_	0.48	0.46	0.43	0.46	0.02
*α*_*r2*_	0.009	0.016	0.010	0.01	3.97e-3
*K*_*z*_	0.07	0.10	0.07	0.08	0.02

Model outputs were matched to target data for 90, 100 and 110% of resting sarcomere length.

### Reduction of model complexity for the Niederer-model and the Land-model

#### ***Parameter identifiability analysis for the Land-model***

**Figure 11:**
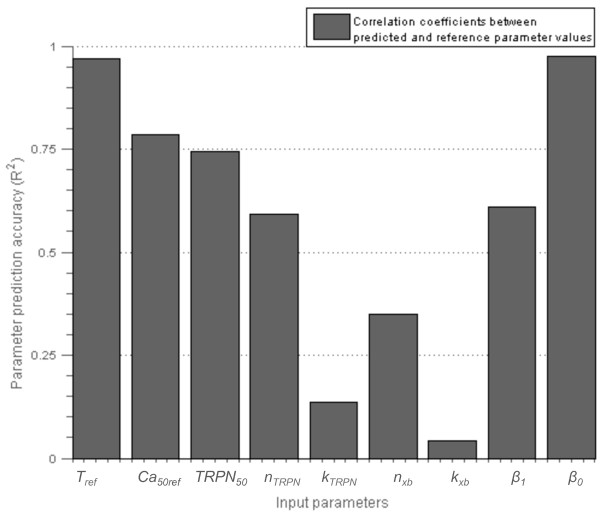
**Results from the parameter identifiability analysis of the Land-model.** Prediction accuracies (test set validated using 33% of the simulations as an independent test set) for the Land-model parameters using inverse HC-PLSR metamodelling with 8 clusters.

In order to identify a reduced version of the Land-model, a LHD of 500 simulations were made with the parameter ranges given in Table 5 for the nine length-dependence parameters of the Land-model. An inverse metamodel was made in the same way as for the Niederer-model, and the test set parameter prediction accuracies achieved are shown in Figure 11. The results in Figure 11 show that only *T*_*ref*_ and *β*_*0*_ had *R*^*2*^-values above 0.8, but also *Ca*_*50ref*_ and *TRPN*_*50*_ had *R*^*2*^-values above 0.7, which is a reasonably good prediction accuracy considering the large span in parameter values utilised here. *n*_*TRPN*_ and *β*_*0*_ had *R*^*2*^-values around 0.6. Hence, most of the parameters from the Land-model could be constrained by the output metrics considered. However, *k*_*TRPN*_, *n*_*xb*_ and *k*_*xb*_ were not as well constrained, having *R*^*2*^-values below 0.5. Hence, the possibility for reducing the model complexity by making a steady state approximation by increasing *k*_*TRPN*_ and *k*_*xb*_ to 10 times the default value was analysed as described below. The low sensitivity to *n*_*xb*_ may be explained by the coupling to *n*_*TRPN*_, which was illustrated in [16]. The effects of removing this parameter by setting it to 1 are analysed below.

#### ***Model reduction***

##### 

**Reduction of the Niederer-model** The values of *α*_*r2*_ in Table 6 are close to zero, and according to the analysis above the Niederer-model has low sensitivity to this parameter. Hence, we tested whether the model can be simplified by giving this parameter the value zero. This gives *K*_*1*_ = 0, *K*_*2*_ = 0 (see Additional file 1), and thereby a simplified equation for *z*_*Max*_. The parts of the equation system containing the parameters *n*_*r*_ and *K*_*z*_ would then also be zero, making these parameters redundant. A new parameter fitting was therefore carried out, starting from the same initial parameter ranges as in the first parameter fitting, but now with *α*_*r2*_ = 0 in all parameter sets. The same parameter fitting procedure as described above was used, and four parameter sets (Table 7) were found to give values of the output metrics close to the target values. Comparison of the parameter sets in Tables 6 and 7 shows that the values are relatively similar for most parameters. Hence, two separate parameter fittings identified the same parameter space region, giving confidence in the parameter estimates.

**Table 7:** **Niederer-model parameter values corresponding to the model output values closest to the target (*α***_***r2***_ **= 0)**

**Parameter**	**Parameter set 1**	**Parameter set 2**	**Parameter set 3**	**Parameter set 4**	**Mean value**	**Standard deviation**
*Ca*_*50ref*_	3.51e-04	3.45e-04	3.45e-04	3.71e-04	3.53e-04	1.23e-05
*k*_*refoff*_	0.11	0.12	0.08	0.14	0.11	0.03
*k*_*on*_	231.4	268.2	240.2	294.0	258.4	28.4
*β*_*0*_	0.66	0.28	0.92	0.71	0.64	0.26
*β*_*1*_	-1.33	-1.34	-1.24	-1.47	-1.35	0.10
*γ*	3.73	4.56	4.61	4.29	4.30	0.41
*nH*	11.31	12.50	14.22	11.70	12.43	1.29
*T*_*ref*_	128.3	126.2	113.4	104.1	118.0	11.3
*α*_*0*_	0.03	0.02	0.04	0.05	0.04	0.01
*α*_*r1*_	0.31	0.28	0.38	0.35	0.33	0.05

Model outputs were matched to target data for 90, 100 and 110% of resting sarcomere length.

**Figure 12:**
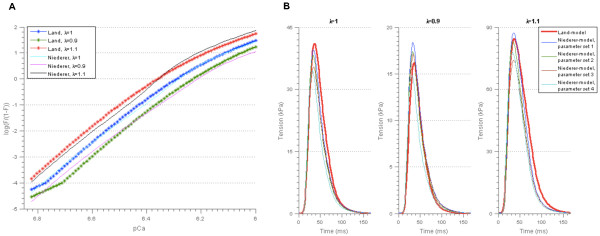
**Resulting model outputs for the reduced version of the Niederer-model. A)** Force-pCa relationship for parameter set 2 in Table 7, found using *α*_*r2*_ = 0. The force-pCa relationships for the remaining parameter sets in Table 7 are shown in Additional file 3: Figure A3.2 and Figure A3.3. **B)** Tension transients for all parameter sets in Table 7, found using *α*_*r2*_ = 0. The parameter λ represents the cell length relative to the resting cell length.

The force-pCa relationship for parameter set 2 in Table 7, which gave the best match to the Land-model outputs, and the tension transients for all parameter sets in Table 7 are shown in Figure 12. The force-pCa relationships for the remaining parameter sets in Table 7 are shown in Additional file 3: Figure A3.2 and Figure A3.3. Our results therefore indicate that it is possible to reduce the Niederer-model by setting *α*_*r2*_ to zero while keeping the same model behaviour.

For this reduced model version, the parameters could be constrained to a standard deviation of on average 14.6% of the mean values over the succeeding parameter sets, as compared to 17.4% for the original model version. This is not a very large decrease in the spread of resulting parameter sets, but this model reduction process has clear advantages in terms of ultimately increasing the capacity to derive physiological insight from the model behaviour and identification of feasible measurements to make in order to constrain parameters.

##### 

**Figure 13:**
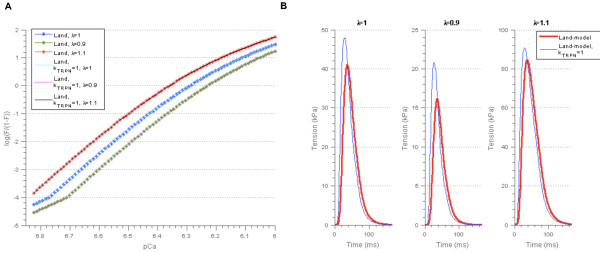
**Resulting model outputs for the Land-model with *k***_***TRPN***_ **= 1. A)** Force-pCa relationship using *k*_*TRPN*_ = 1. **B)** Tension transients achieved with *k*_*TRPN*_ = 1. The parameter *λ* represents the cell length relative to the resting cell length.

**Figure 14:**
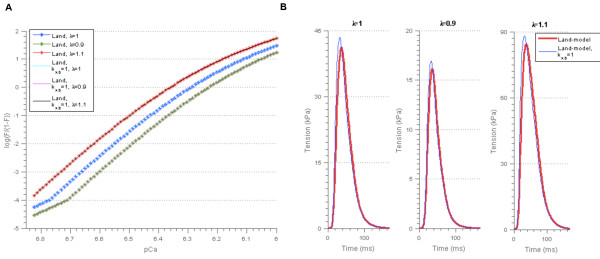
**Resulting model outputs for the Land-model with *k***_***xb***_ **= 1. A)** Force-pCa relationship using *k*_*xb*_ = 1. **B)** Tension transients achieved with *k*_*xb*_ = 1. The parameter *λ* represents the cell length relative to the resting cell length.

**Reduction of the Land-model** The parameter identifiability analysis indicated that the Land-model had relatively low sensitivity to the parameters *k*_*TRPN*_, *n*_*xb*_ and *k*_*xb*_ in the part of the simulation space analysed here. These three parameters were therefore successively given the value 1, while all the other parameters were kept at the default values, and simulations were run in order to analyse the consequences these changes had for the model outputs. Giving these parameters the value 1 simplifies the equation system for the Land-model (see Additional file 1). Setting *n*_*xb*_ = 1 led to relatively large changes in model behaviour (results not shown), as expected considering the importance of thin filament cooperativity. However, setting *k*_*TRPN*_ = 1 or *k*_*xb*_ = 1 had only relatively small consequences for the behaviour; the force-pCa relationships were identical to the default output, and the tension transients were still within the measurement error compared to the default tension transients (see Figures 13 and 14). Hence, this indicates that it is possible to speed up these components of the Land-model to near steady state by setting *k*_*TRPN*_ = 1 or *k*_*xb*_ = 1 while keeping approximately the same model behaviour. This result was probably caused by the fact that the measured time to peak is relatively low for mouse, giving these two parameters undefined upper bounds given the metrics included in this analysis (both parameters have a well-defined lower bound of zero). Setting both to 1 simultaneously caused the time to peak to be too low compared to the measured data, as expected. This indicates that it is difficult to identify the rate-limiting step using the metrics included in this study, something that is consistent with the coupling of *k*_*TRPN*_ and *k*_*xb*_ found previously [16].

## Discussion

In this study, we have presented and demonstrated the value of a generic and robust methodology for combined parameter fitting and analysis of model mechanisms. To demonstrate this method, we have adjusted the parameters of the Niederer-model to fit data for mouse at 37°C. We also succeeded in finding reduced versions of both the Land-model and the Niederer-model through comparison of model alternatives and fitting of reduced model versions to measured data. Our results indicate that this is an effective approach for comparing model alternatives and reducing models to the minimum complexity replicating measured data.

In our analysis we make the assumption that the equations capture the salient first order dynamics of our system of interest. Both models applied here are biophysically based. By understanding the relationships between the parameters and model predictions, we gain further insight into the regulation and physiology of our system. The Niederer-model has two relaxation terms, but setting *α*_*r2*_ to zero leaves only one relaxation term. The omitted relaxation term was designed to fit rapid relaxation rates following a step change in calcium due to the activation of a calcium chelator. However, our analysis shows that a simpler model suffices for contraction under conditions of regular changes in calcium, which includes most physiological conditions. The Land-model starts with only one relaxation term, so it cannot be removed. Setting the parameters *k*_*TRPN*_ or *k*_*xb*_ to 1 are approximations for very fast or near steady-state kinetics.

The fitting pipeline includes an implicit sensitivity analysis and analysis of parameter identifiability, making it suitable for testing hypotheses for model reduction. Hence, an advantage of this method compared to alternative methods is that it not only provides the parameter values, but also gives an estimate of the identifiability of parameters and the uncertainty in the parameter estimates through both the range of values in the feasible parameter sets and the ability of the inverse metamodel to predict the different parameters. Combining the analysis of model mechanisms with parameter fitting makes it possible to automatically detect how the behaviour of the model as well as the parameter identifiability changes as a consequence of moving to different parts of the parameter space, and whether adjusting certain parameters makes other parameters or model components redundant.

### Sensitivity analysis

Biological models typically contain numerous output metrics resulting from large sets of coupled equations, and complex covariance patterns often exist between the outputs. Choosing the measurements to make in order to constrain biological parameters thus requires sensitivity analyses and parameter fitting methodologies that can take numerous output variables into account simultaneously and evaluate the impact of parameter value perturbations on the entire model system. Regression-based sensitivity, as used here, is based on deriving a selection of data points by experimental design or semi-random sampling, and analysing the resulting input–output relations using regression [36]. The regression coefficients then provide direct measures of the impact of variations of the individual inputs on the output. Most regression-based sensitivity analyses published are based on relatively simple linear models fitted by OLS Regression [37]. In this study, the sensitivity analysis was based on classical PLSR metamodelling due to its ability to handle linearly dependent regressor variables, several response variables simultaneously and to utilise inter-correlations between the response variables for regression model stabilisation.

Metamodelling has been widely used in e.g. engineering, for speed-up of computations, sensitivity analysis and uncertainty assessment [37], and recently, multivariate metamodelling using PLSR [25-28,30,38] and HC-PLSR [5,6,29] has been shown to be effective for analysis of the complex, nonlinear input–output relationships of biological models. Classical PLSR metamodels, where the model outputs are predicted as functions of the input parameters, are useful for sensitivity analysis and analysis of interactions between input parameters and covariance patterns between multiple model outputs [29].

Several alternatives to regression-based sensitivity analysis exist, such as rank transformation, first- and second order reliability algorithms (FORM and SORM) and variance-based methods [36]. Rank transformation is an alternative to conventional regression-based sensitivity analysis in cases where the input–output relations are monotonically nonlinear, while reliability algorithms are used in cases where the primary focus is on a particular mode of failure of the system rather than the entire spectrum of possible outcomes. Variance-based methods, such as Sobol's method [39], use Analysis of Variance (ANOVA)-type decomposition of the output function into a polynomial expression including cross-terms between the input parameters. Partial variances are computed from each of the terms in the decomposition, and the sensitivity of each term is defined as the partial variance divided by the total output variance. However, these methods concentrate on the effects on one output variable at a time, and are therefore not as useful for analysis of biological systems that typically contain intricate feedback loops.

### Parameter fitting

As described above, in order to re-parameterise the Niederer-model, we used a combination of inverse metamodelling [5,8], predicting the input parameter values directly from the model output metrics, and iterative zooming into the relevant region of the parameter space based on a look-up approach. However, numerous alternative methods exist to fit model parameters from measured data. Optimisation of the parameter values based on simplex optimisation [12] is a widely used approach. However, the results become unreliable when many parameters are required to be fitted simultaneously, and the most common approach is to fit a few parameters at a time. The result from simplex optimisation is highly dependent on the starting values used, and this method is thus often not able to find the global optimum. The optimisation itself is computationally non-expensive, but the optimisation might become time consuming if the dynamic model is large, since the optimisation has to be run many times with different starting values to provide reliable results.

In order to compare our method to the more conventional simplex optimisation, we ran optimisations with the Nelder-Mead simplex (direct search) method [40] using the “fminsearch” function in MATLAB^®^ (with default settings). Optimisations were run using 50 different starting values (randomly chosen from the initial design used in our metamodelling-based parameter fitting pipeline), adding a penalty to the cost function value for moving outside the feasible parameter ranges given in Table 4. The cost-function we used was the RMSD between the simulated and reference model outputs (Tables 2 and 3). The RMSD was calculated using output variables that had been scaled by subtracting the mean and dividing by the standard deviation for all model outputs from simulations using the initial experimental design described under “Fitting of the Niederer-model parameters” in the Methods section. None of the optimisations could identify any parameter sets within the feasible ranges producing model outputs replicating the reference data. Even though we used a wide variety of starting values and penalty functions, all optimisations were driven outside the feasible region, and were unable to move back into the feasible region, in spite of the penalty added to the cost function. It therefore seems that with a very complex cost function with many local minima like the one used in this study, our statistical approach is more useful than the simplex optimisation for constraining the model parameters.

Alternative optimisation methods include simulated annealing [13] and Levenberg-Marquart optimisation [14]. These methods generally give more reliable results, and are more likely to find the global optimum. However, they are also significantly more computationally expensive, and are therefore not very suitable for parameter estimation of large, dynamic models. Moreover, neither of these methods or the simplex optimisation provide an increased understanding of the underlying model mechanisms, they result in a parameter estimate only, and the results can often be non-physiological when no constraints on the parameter values are used.

As an alternative, Artificial Neural Network-based methods [41] are computationally non-expensive and can fit input–output relations including several outputs successfully. However, the neural network models often become extremely complex and difficult to interpret. They are also highly dependent on the quality of the data, and since they have the flexibility to adjust to small nuances in the data there is a risk of fitting to noise. Physiological measurements often lack a sufficient signal-to-noise ratio, giving non-robust approximations of the parameter values when these methods are used for parameter estimation. Kalman Filtering [42] and derivative-based methods give an estimate of parameter confidence, but can be computationally expensive, and derivative-based methods may in addition display convergence problems.

Sarkar and Sobie [43] recently published a regression-based approach for constraining free parameters in dynamic models, based on inverting the regression coefficient matrix of a classical metamodel, and using this inverted regression coefficient matrix to predict the parameters from the model outputs. This resembles inverse metamodelling, but in inverse metamodelling the input parameters are predicted directly from the output metrics from simulations using regression, avoiding the need for an invertible (square) regression coefficient matrix. Both the approach presented by Sarkar and Sobie and the inverse metamodelling approach require a non-ambigous (one-to-one) relationship between input parameters and model outputs. This, however, is often not the case for many biologically based models (often referred to as model sloppiness [35]), creating a need for an alternative approach to constrain model parameters. This model sloppiness was also demonstrated in the application in this study, where low parameter identifiability resulted from the initial analysis (Figure 6).

In spite of model sloppiness, inverse metamodelling can effectively identify the direction in the parameter space to move in order to approach measured data in cases where the baseline is far from the target. This can limit the search space compared to what is needed with alternative methods such as simplex optimisation. Without prior knowledge of suitable starting values for the optimisation, a simplex optimisation requires numerous simulations to give reasonable results. In contrast, the inverse metamodelling component of our method effectively guides the design of new simulations towards the most relevant parts of the parameter space, and the search space can thereby be reduced. This can also be achieved with methods like genetic algorithms or Levenberg-Marquardt optimisation. However, these methods provide no implicit, easily interpretable analysis of model mechanisms.

If the inverse metamodel is not calibrated using relevant simulation results, it has the potential to identify an incorrect search direction in the parameter space. However, the look-up process will automatically detect this error, since the closest simulations will then be further from the measurements than in the previous iteration. In such cases, the inverse metamodelling component can be omitted, and the look-up part of the algorithm used alone to guide the design of new simulations. The method often results in a set of possible solutions that can be restricted according to known physiological ranges of the parameters. Accordingly, as new measurements of output metrics or parameters become available, they can further constrain the set of possible solutions. Hence, prior knowledge can easily be taken into account in the procedure. Moreover, other cost-functions can easily be incorporated in the pipeline, in addition to, or instead of, the RMSD calculated in the PCA score space. Hence, a weighting of the output metrics according to, for example, relevance for clinical use can be utilised.

Due to the dependency of the results from each fitting iteration on the previous iteration, there may be other directions in the parameter space that could also give possible solutions. Hence, the parameter space needs to be sampled densely in the initial experimental design to ensure that all possible solutions are found. However, in each iteration the experimental design is extended slightly beyond the ranges of the guideline set. Hence, alternative directions in the parameter space that would allow model outputs replicating the measurements are likely to be found during the procedure. In cases where the target is very far from the output of the baseline parameter set, the method may need numerous simulations to make sure the parameter space is sampled sufficiently and that all possible clusters of feasible solutions are found, but due to the effective identification of a reasonable direction in the parameter space to move by the inverse metamodelling, the method is still likely to be more efficient in most cases than a “brute force” optimisation using, for example the simplex method, with a large number of different starting values. The method gives no clear answer as to when to stop, how many parameter values are enough or how we can know whether we have found all possible clusters/manifolds of solutions, but this is a problem with any parameter estimation method. Likewise, if the data used to fit the parameters does not cover the complete space of system behaviour, the model parameters will not be constrained by the data, which also means that the model is too complex for the data it is being used to understand. This is true for all models and parameterisation methods.

### Model reduction

Parameterising cardiac cell models in a whole-organ context is important for multi-scale modelling and ultimately for clinical use of the models, and requires the ability to control and foresee the whole-organ consequences of variations in cell-level model parameters. This makes it easier to determine how to pass on parameters between the scales, and eases the parameterisation of the cell-models in a whole-organ context. This again requires compact cell models with relatively few parameters and equations for which overview of the input–output relationships can be easily gained. By reducing models to a minimum number of parameters and equations, using detailed biophysical data we can reduce the number of free parameters that can then be efficiently fitted when these cellular models are embedded within whole organ models and fitted to compatible data. In many cases, and in particular clinical contexts, only whole organ data will be available. Consequently, there is a need for efficient comparison of model alternatives in order to find the most reduced version that is able to replicate experimental measurements. For biochemical reaction networks, several methods have been developed for reducing the networks to the minimal complexity required [44]. We present here a generic framework for combined sensitivity analysis, parameter identifiability analysis, parameter fitting and model reduction, which can be applied to all types of deterministic models generating a set of outputs from a set of input parameters.

Our results indicate that the presented approach is effective for model reduction and automatic updating of models according to new measurements, allowing identification of models that are more specific to e.g. certain species, temperatures or individuals. This is likely to be important in large modelling initiatives like the Physiome project (physiomeproject.org), since compact cell models can be more confidently and effectively applied as parts of large multi-scale whole organ models. We therefore believe that the presented methodology will be of great value for future model development, including the search for patient-specific or patient group-specific parameter values, something that is likely to highly increase the clinical applicability of models.

## Conclusions

We have presented a new method for parameter estimation, which combines parameter fitting in relation to measured data and analysis of the mechanisms of the model system. The pipeline contains an implicit analysis of the model sensitivity and the parameter identifiability for model reduction. Using our approach, different model alternatives can be compared, allowing effective analysis of the consequences of introducing changes to the models and identification of redundant model components that can be omitted without affecting the fit to measured data. We have applied the methodology to show that we can make two alternative model frameworks for cardiac contraction give the same outputs, and that we can generate reduced versions of both these models using this approach. We show that despite model sloppiness, inverse metamodelling can identify a reasonable direction in the parameter space to move in order to approach measured data. Combined with a look-up of simulations in the proximity of the measured data and iterative generation of new experimental designs, this provides an accurate and effective approach for constraining model parameters.

The presented parameter fitting pipeline can effectively fit numerous parameters simultaneously, and through the iterative generation of new experimental designs for simulations, the method provides an overview of the spread of possible solutions, as well as possible clusters of suitable parameter values. This indicates the ability of a set of output metrics to constrain the parameters and gives an estimate of the uncertainty in the parameter estimates. In this study we showed that the Niederer-model parameters could be constrained to a standard deviation of on average 17.4% of the mean values over the succeeding parameter sets. This was decreased to 14.6% for the equivalent reduced model. As new measurements become available, these can be incorporated to further constrain parameter values.

Given measured data for a number of patients in a clinical context, this methodology can also be used to find sets of parameter values replicating the measured data for each patient, allowing identification of clusters in the parameter space corresponding to different patients or patient groups for personalised medicine. Similarly, clusters of parameter values for different species, different measurement conditions etc. can be identified. The presented method thus has a clear potential in both multi-scale model development and clinical use of models.

## Competing interests

The authors declare that they have no competing interests.

## Authors’ contributions

KT contributed to conception of the study and design of the computer experiments, wrote the MATLAB^®^ code for the parameter fitting pipeline, performed the computer simulations, analysed the data and wrote the paper. SAN contributed to conception of the study and to writing of the paper. SL participated in designing the computer experiments and in debugging of the MATLAB^®^ code. NPS contributed to conception and coordination of the study and to writing of the paper. All authors read and approved the final manuscript.

## Supplementary Material

Additional file 1 **Description of the contraction models.** A1.1. Length-dependence equations of the Land-model. A1.2. Length-dependence equations of the Niederer-model. Click here for file

Additional file 2 Description of Hierarchical Cluster-based PLSR. Click here for file

Additional file 3 **Additional figures.** Figure A3.1. Force-pCa relationships for parameter sets 2 and 3 in Table 6. Figure A3.2. Force-pCa relationships for parameter set 1 and 3 in Table 7. Figure A3.3. Force-pCa relationships for parameter set 4 in Table 7. Click here for file

## References

[B1] Nickerson DP Hunter PJ The Noble cardiac ventricular electrophysiology models in CellML Prog Biophys Mol Biol 2006 90 346 359 15979694 10.1016/j.pbiomolbio.2005.05.007

[B2] Smith NP Crampin EJ Niederer SA Bassingthwaighte JB Beard DA Computational biology of cardiac myocytes: proposed standards for the physiome J Exp Biol 2007 210 Pt 9 1576 1583 17449822 10.1242/jeb.000133 PMC2866297

[B3] Niederer SA Fink M Noble D Smith NP A meta-analysis of cardiac electrophysiology computational models Exp Physiol 2009 94 486 495 19139063 10.1113/expphysiol.2008.044610

[B4] Hunter PJ Borg TK Integration from proteins to organs: the Physiome Project Nat Rev Mol Cell Biol 2003 4 237 243 12612642 10.1038/nrm1054

[B5] Tøndel K Indahl UG Gjuvsland AB Omholt SW Martens H Multi-way metamodelling facilitates insight into the complex input–output maps of nonlinear dynamic models BMC Syst Biol 2012 6 88 22818032 10.1186/1752-0509-6-88 PMC3483253

[B6] Tøndel K Indahl UG Gjuvsland AB Vik JO Hunter P Omholt SW Martens H Hierarchical Cluster-based Partial Least Squares Regression is an efficient tool for metamodelling of nonlinear dynamic models BMC Syst Biol 2011 5 90 21627852 10.1186/1752-0509-5-90 PMC3127793

[B7] Isaeva J Martens M Sæbø S Wyller JA Martens H The modelome of line curvature: Many nonlinear models approximated by a single bi-linear metamodel with verbal profiling Phys Nonlinear Phenom 2012 241 877 889

[B8] Isaeva J Sæbo S Wyller JA Nhek S Martens H Fast and comprehensive fitting of complex mathematical models to massive amounts of empirical data Chemometr Intell Lab 2012 117 13 21

[B9] Isaeva J Sæbø S Wyller JA Wolkenhauer O Martens H Nonlinear modelling of curvature by bi-linear metamodelling Chemometr Intell Lab 2012 117 2 12

[B10] Pearson K On lines and planes of closest fit to systems of points in space Philos Mag 1901 2 559 572

[B11] Jolliffe IT A Note on the Use of Principal Components in Regression J R Stat Soc: Ser C: Appl Stat 1982 31 300 303

[B12] Murty KG Linear Programming 1983 New York, USA: John Wiley & Sons, Inc

[B13] Kirkpatrick S Gelatt CD Vecchi MP Optimization by Simulated Annealing Science 1983 220 671 680 17813860 10.1126/science.220.4598.671

[B14] Marquardt DW An Algorithm for Least-Squares Estimation of Nonlinear Parameters J Soc Ind Appl Math 1963 11 431 441

[B15] Niederer SA Hunter PJ Smith NP A Quantitative Analysis of Cardiac Myocyte Relaxation: A Simulation Study Biophys J 2006 90 1697 1722 16339881 10.1529/biophysj.105.069534 PMC1367320

[B16] Land S Niederer SA Aronsen JM Espe EKS Zhang L Louch WE Sjaastad I Sejersted OM Smith NP An analysis of deformation-dependent electromechanical coupling in the mouse heart J Physiol 2012 590 Pt 18 4553 4569 22615436 10.1113/jphysiol.2012.231928 PMC3477757

[B17] Land S **An integrative framework for computational modelling of cardiac electromechanics in the mouse** 2013 UK: Doctoral Thesis, University of Oxford

[B18] Gordon AM Homsher E Regnier M Regulation of contraction in striated muscle Physiol Rev 2000 80 853 924 10747208 10.1152/physrev.2000.80.2.853

[B19] Rice JJ Wang F Bers DM de Tombe PP Approximate model of cooperative activation and crossbridge cycling in cardiac muscle using ordinary differential equations Biophys J 2008 95 2368 2390 18234826 10.1529/biophysj.107.119487 PMC2517033

[B20] Hunter PJ McCulloch AD ter Keurs HE Modelling the mechanical properties of cardiac muscle Prog Biophys Mol Biol 1998 69 289 331 9785944 10.1016/s0079-6107(98)00013-3

[B21] McKay MD Los ASL Beckman RJ Conover WJ Comparison the three methods for selecting values of input variable in the analysis of output from a computer code Technometrics 1979 21 239 245

[B22] Land S Louch WE Niederer SA Aronsen JM Christensen G Sjaastad I Sejersted OM Smith NP Beta-adrenergic stimulation maintains cardiac function in Serca2 knockout mice Biophys J 2013 104 1349 1356 23528094 10.1016/j.bpj.2013.01.042 PMC3602781

[B23] MATLAB® 2012 Natick, Massachusetts, USA: The MathWorks, Inc

[B24] Lawson CL Hanson RJ Solving Least Squares Problems 1974 New Jersey, USA: Society for Industrial and Applied Mathematics, Prentice-Hall, Inc.

[B25] Martens M Martens H Partial Least Squares regression Stat Proced Food Res JR Piggott Ed 1986 London: Elsevier Applied Sciences 293 360

[B26] Martens H Næs T Multivariate Calibration 1989 Chichester, UK: John Wiley and Sons

[B27] Martens H Martens M Multivariate Analysis of Quality: An Introduction 2001 1 Chichester, UK: John Wiley & Sons Ltd

[B28] Wold S Martens H Wold H The multivariate calibration method in chemistry solved by the PLS method Lect Notes Math Matrix Pencils 1983 Heidelberg: Springer-Verlag 286 293

[B29] Tøndel K Vik JO Martens H Indahl UG Smith N Omholt SW Hierarchical multivariate regression-based sensitivity analysis reveals complex parameter interaction patterns in dynamic models Chemometr Intell Lab 2013 120 25 41

[B30] Tøndel K Gjuvsland AB Måge I Martens H Screening design for computer experiments: Metamodelling of a deterministic mathematical model of the mammalian circadian clock J Chemometr 2010 24 738 747

[B31] Bezdek JC Pattern Recognition with Fuzzy Objective Function Algorithms 1981 Norwell, USA: Kluwer Academic Publishers

[B32] Berget I Mevik B-H Næs T New modifications and applications of fuzzy C-means methodology Comput Stat Data Anal 2008 52 2403 2418

[B33] Næs T Kubberød E Sivertsen H Identifying and interpreting market segments using conjoint analysis Food Qual Prefer 2001 12 133 143

[B34] Næs T Isaksson T Splitting of calibration data by cluster analysis J Chemometr 1991 5 49 65

[B35] Gutenkunst RN Waterfall JJ Casey FP Brown KS Myers CR Sethna JP Universally Sloppy Parameter Sensitivities in Systems Biology Models PLoS Comput Biol 2007 3 e189 10.1371/journal.pcbi.0030189 PMC2000971 17922568

[B36] Cacuci DG Ionescu-Bujor M Navon IM Sensitivity and Uncertainty Analysis: Applications to Large-Scale Systems Vol 2 2005 1 Boca Raton, USA: CRC Press

[B37] Kleijnen JPC Design and Analysis of Simulation Experiments 2007 1 New York, USA: Springer

[B38] Vik JO Gjuvsland AB Li L Tøndel K Niederer SA Smith N Hunter PJ Omholt SW Genotype-phenotype map characteristics of an in silico heart cell Front Physiol 2011 2 106 22232604 10.3389/fphys.2011.00106 PMC3246639

[B39] Sobol IM Global sensitivity indices for nonlinear mathematical models and their Monte Carlo estimates Math Comput Simul 2001 55 271 280

[B40] Nelder JA Mead R A Simplex Method for Function Minimization Comput J 1965 7 308 313

[B41] El Tabach E Lancelot L Shahrour I Najjar Y Use of artificial neural network simulation metamodelling to assess groundwater contamination in a road project Math Comput Model 2007 45 766 776

[B42] Hamilton J The Kalman Filter Time Ser Anal 1994 13 New Jersey, USA: Princeton University Press

[B43] Sarkar AX Sobie EA Regression Analysis for Constraining Free Parameters in Electrophysiological Models of Cardiac Cells PLoS Comput Biol 2010 6 10.1371/journal.pcbi.1000914 PMC2932676 20824123

[B44] Radulescu O Gorban AN Zinovyev A Noel V Reduction of dynamical biochemical reactions networks in computational biology Front Gene 2012 3 131 10.3389/fgene.2012.00131 PMC3400272 22833754

