# Supplementary material for: Insight into model mechanisms through automatic parameter fitting: a new methodological framework for model development
Source: BMC Syst Biol. 2014 May 20;8:59. doi: 10.1186/1752-0509-8-59 (PMC4078362; doi:10.1186/1752-0509-8-59)
Supplement: Additional file 1 — Description of the contraction models. A1.1. Length-dependence equations of the Land-model. A1.2. Length-dependence equations of the Niederer-model. [file 1752-0509-8-59-S1.pdf]

Additional file 1. Description of the contraction models

A1.1. Length-dependence equations of the Land-model

In the Land-model, the calcium sensitivity is given by

$$[Ca^{2+}]_{50} = Ca_{50ref}(1 + \beta_1(\lambda - 1)) \tag{A1.1}$$

where λ is the extension ratio, i.e. the sarcomere length relative to the resting sarcomere length.

The dynamics of the fraction of regulatory troponin C sites with bound calcium is given by

$$\frac{dTRPN}{dt} = k_{TRPN}\left(\left(\frac{[Ca^{2+}]_i}{[Ca^{2+}]_{50}}\right)^{n_{TRPN}}(1 - TRPN) - TRPN\right) \tag{A1.2}$$

The dynamics of the fraction of available crossbridges cycling is given by

$$\frac{dXB}{dt} = k_{xb}\left(permtot\ (1 - XB) - \frac{1}{permtot}XB\right) \tag{A1.3}$$

where

$$permtot = \sqrt{\left(\frac{TRPN}{TRPN_{50}}\right)^{n_{xb}}} \tag{A1.4}$$

The influence of filament overlap on tension is taken into account by

$$h(\lambda) = max\ (0, h'\ \ (min(\lambda, 1.2))) \tag{A1.5}$$

where

$$h'(\lambda) = 1 + \beta_0(\lambda + \min(\lambda, 0.87) - 1.87) \tag{A1.6}$$

When the velocity dependence is not taken into account, the normalised force then becomes

$$F_n = h(\lambda) \times XB \tag{A1.7}$$

and the active tension is given by

$$T_a = T_{ref} \times F_n \tag{A1.8}$$

A1.2. Length-dependence equations of the Niederer-model

Also in the Niederer-model, the Ca-sensitivity, $[Ca^{2+}]_{50}$, is given by Equation (A1.1). The dynamics of the concentration of Ca^{2+} bound to Troponin C site II, $[Ca^{2+}]_{Trpn}$, is given by

$$\frac{d[Ca^{2+}]_{Trpn}}{dt} = k_{on}[Ca^{2+}]_i\left([Ca^{2+}]_{TrpnMax} - [Ca^{2+}]_{Trpn}\right) - k_{off}\left(T\right)[Ca^{2+}]_{Trpn} \qquad \text{(A1.9)}$$

where $[Ca^{2+}]_{TrpnMax}$ is the maximum concentration of ions that can bind to Troponin C site II, $[Ca^{+2}]_i$ is the concentration of free Ca^{2+}, T is the tension and

$$k_{off} = k_{refoff}\left(1 - \frac{T}{\gamma T_{ref}}\right) \qquad \text{(A1.10)}$$

Combined with

$$T_{0Max} = T_{ref}\left(1 + \beta_0\left(\lambda - 1\right)\right) \qquad \text{(A1.11)}$$

where T_{0Max} is the maximum tension at full activation for a given sarcomere length, we get

$$[Ca^{2+}]_{Trpn50} = [Ca^{2+}]_{TrpnMax}\frac{[Ca^{2+}]_{50}}{[Ca^{2+}]_{50} + \frac{k_{refoff}}{k_{on}}\left(1 - \frac{\left(1 + \beta_0\left(\lambda - 1\right)\right)\cdot 0.5}{\gamma}\right)} \qquad \text{(A1.12)}$$

The dynamics of the fraction of actin sites available for crossbridge binding is given by

$$\frac{dz}{dt} = \alpha_0\left(\frac{[Ca^{2+}]_{Trpn}}{[Ca^{2+}]_{Trpn50}}\right)^{nH}\left(1 - z\right) - \alpha_{r1}z - \alpha_{r2}\frac{z^{n_r}}{z^{n_r} + K_Z^{n_r}} \qquad \text{(A1.13)}$$

which gives

$$z_{Max} = \frac{\dfrac{\alpha_0}{\left(\dfrac{[Ca^{2+}]_{Trpn50}}{[Ca^{2+}]_{TrpnMax}}\right)^{nH}} - K_2}{\alpha_{r1} + K_1 + \dfrac{\alpha_0}{\left(\dfrac{[Ca^{2+}]_{Trpn50}}{[Ca^{2+}]_{TrpnMax}}\right)^{nH}}} \qquad \text{(A1.14)}$$

where z_{Max} is the maximum fraction of available actin sites at a given sarcomere length, and

$$K_2 = \alpha_{r2} \frac{z_p^{\,n_r}}{z_p^{\,n_r} + K_Z^{\,n_r}} \left(1 - \frac{z_p}{\left(z_p^{\,n_r} + K_Z^{\,n_r} \right)} \right) \tag{A1.15}$$

$$K_1 = \frac{\alpha_{r2} z_p^{\,n_r - 1} n_r K_Z^{\,n_r}}{\left(z_p^{\,n_r} + K_Z^{\,n_r} \right)^2} \tag{A1.16}$$

Isometric tension is then defined as

$$T_0 = T_{0Max} \frac{z}{z_{Max}} = T_{ref} \left(1 + \beta_0 \left(\lambda - 1 \right) \right) \cdot \frac{z}{z_{Max}} \tag{A1.17}$$
