# Supplementary material for: Insight into model mechanisms through automatic parameter fitting: a new methodological framework for model development
Source: BMC Syst Biol. 2014 May 20;8:59. doi: 10.1186/1752-0509-8-59 (PMC4078362; doi:10.1186/1752-0509-8-59)
Supplement: Additional file 2 — Description of Hierarchical Cluster-based PLSR. [file 1752-0509-8-59-S2.pdf]

Additional file 2. Description of Hierarchical Cluster-based PLSR

Hierarchical Cluster-based Partial Least Squares Regression (HC-PLSR) works by first generating a global PLSR model based on all observations, relating the regressors (*X*) to the response (*Y*). A chosen number of score vectors are then used to cluster the observations into groups using fuzzy *C*-means (FCM) clustering [31–34]. Regional PLSR models are then made within each cluster. Polynomial regression can be used both in the global and the regional regression models. For prediction, PLSR score vectors for new observations are calculated based on the new *X*-data, and these predicted score vectors are then used for classifying the observations into the different calibration set clusters. HC-PLSR predictions of the response can then be done either by choosing the most probable regional regression model (cluster), or by using a weighted sum of the regional regression models based on estimated cluster membership probabilities. A flow chart of the HC-PLSR algorithm is given in Figure A2.1.

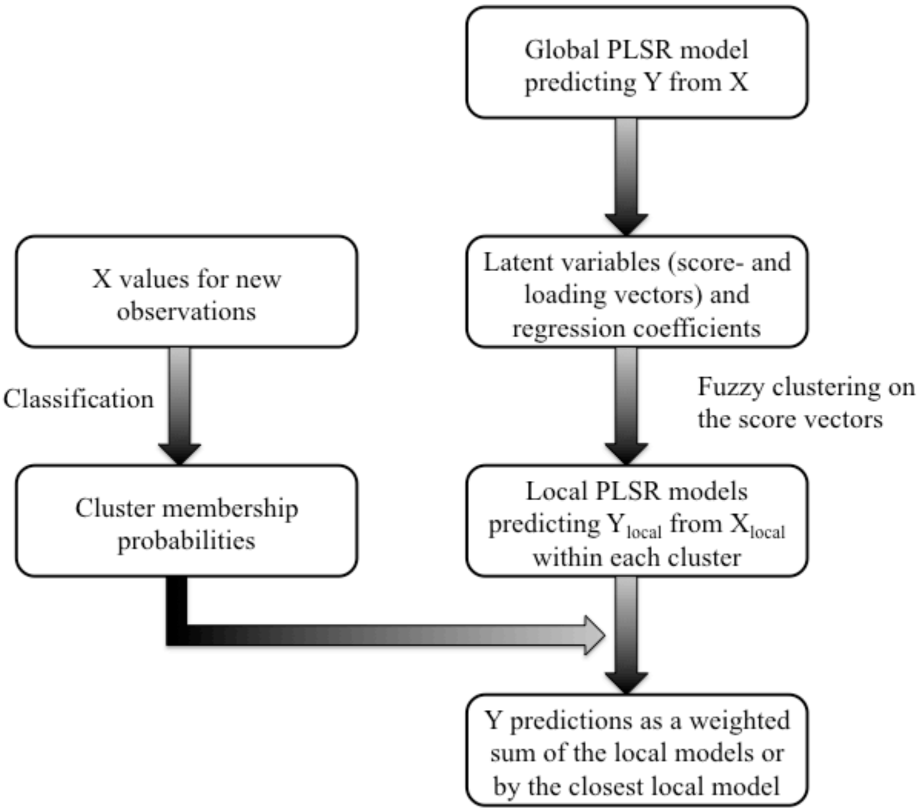

Figure A2.1. Illustration of the Hierarchical Cluster-based Partial Least Squares Regression (HC-PLSR) method.
