# Supplementary material for: Insight into model mechanisms through automatic parameter fitting: a new methodological framework for model development
Source: BMC Syst Biol. 2014 May 20;8:59. doi: 10.1186/1752-0509-8-59 (PMC4078362; doi:10.1186/1752-0509-8-59)
Supplement: Additional file 3 — Additional figures. Figure A3.1. Force-pCa relationships for parameter sets 2 and 3 in Table 6. Figure A3.2. Force-pCa relationships for parameter set 1 and 3 in Table 7. Figure A3.3. Force-pCa relationships for parameter set 4 in Table 7. [file 1752-0509-8-59-S3.pdf]

Additional file 3. Additional figures

Parameter set 2:

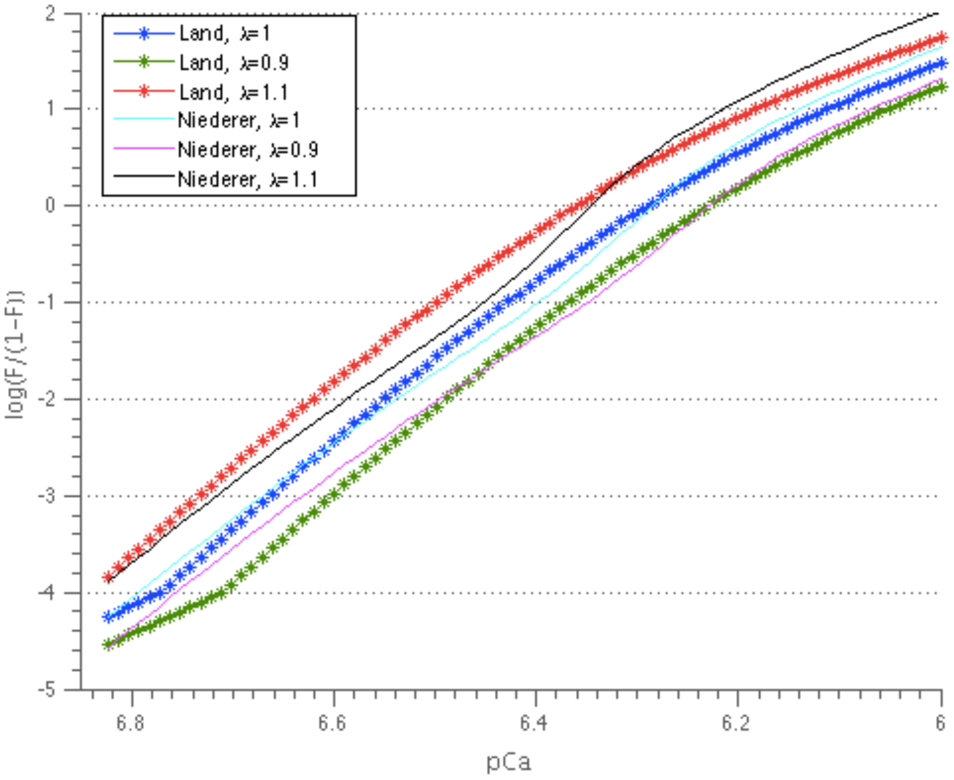

Parameter set 3:

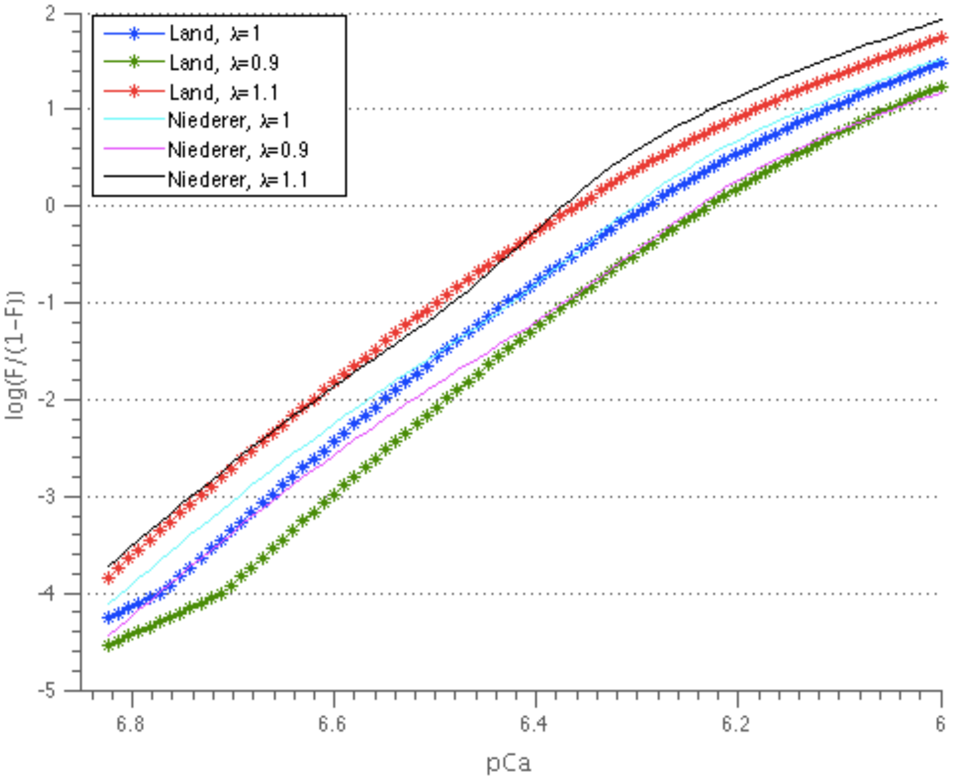

Figure A3.1. Force-pCa relationships for parameter sets 2 and 3 in Table 6.

Parameter set 1:

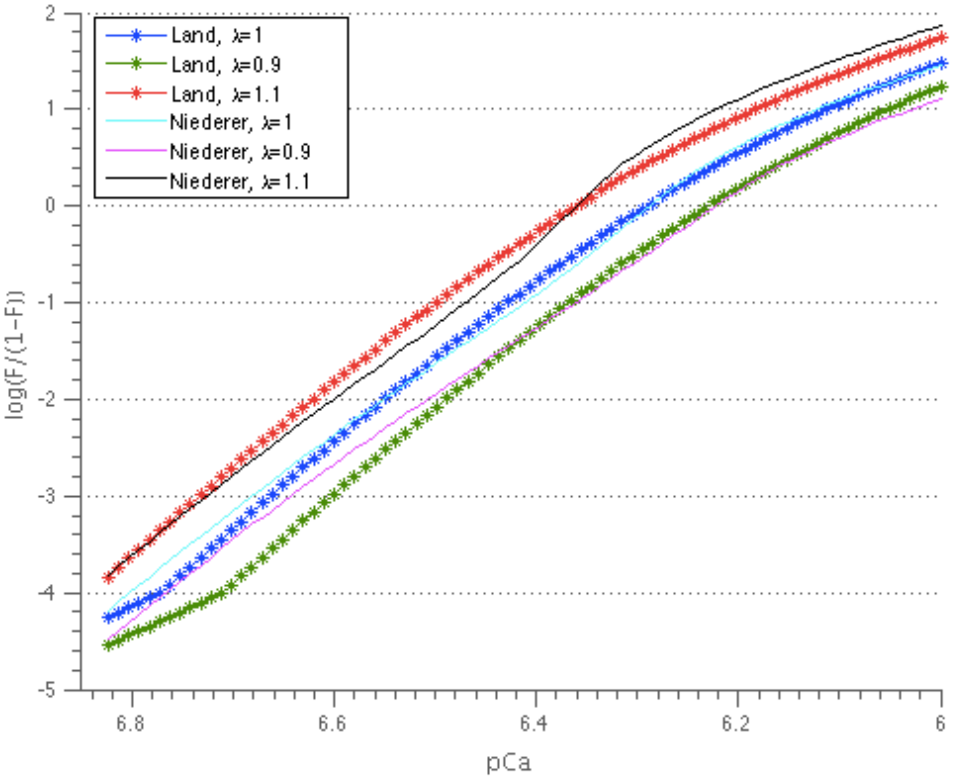

Parameter set 3:

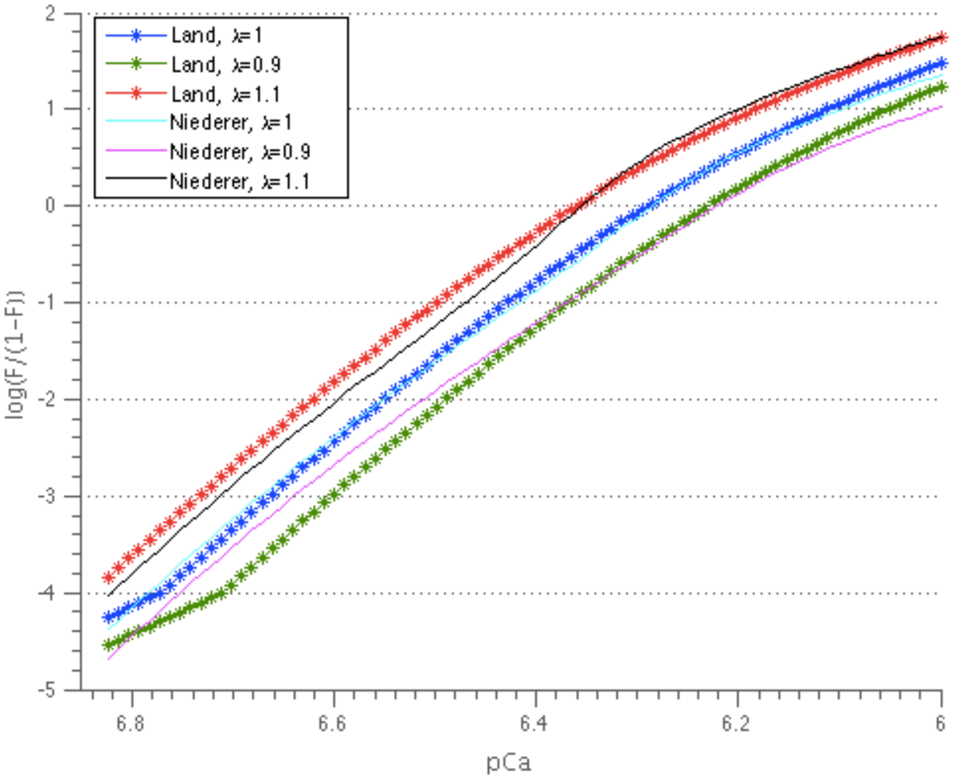

Figure A3.2. Force-pCa relationships for parameter set 1 and 3 in Table 7.

Parameter set 4:

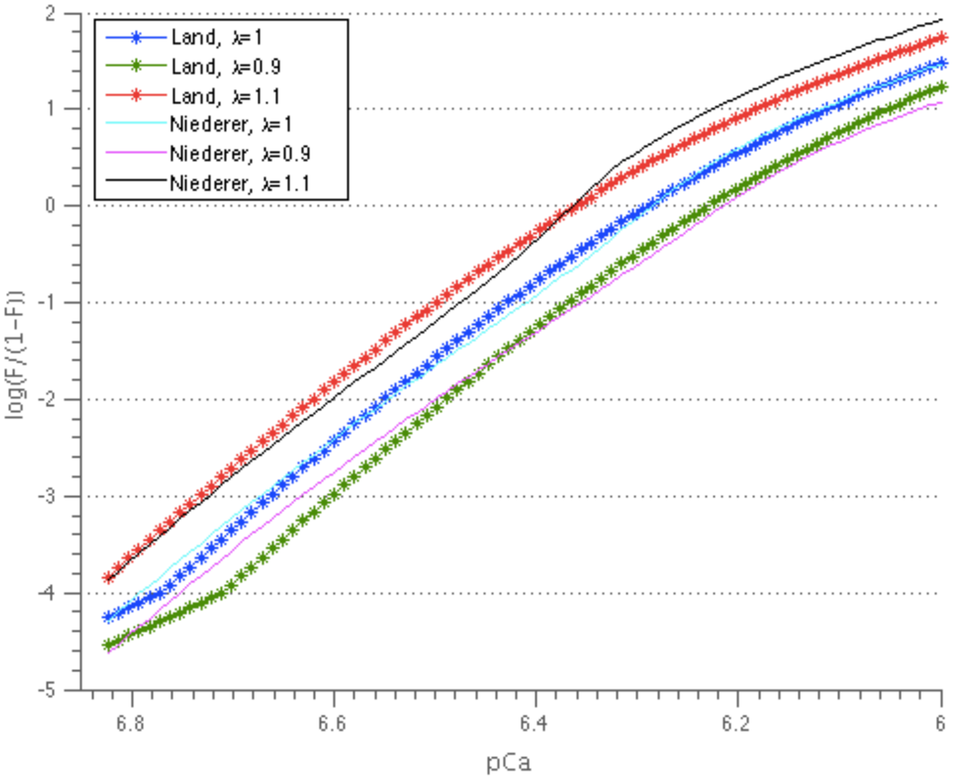

Figure A3.3. Force-pCa relationships for parameter set 4 in Table 7.
